# Quantitation of cytopathic honey bee viruses using the viral plaque assay method

**DOI:** 10.1099/jgv.0.002304

**Published:** 2026-07-28

**Authors:** Alexander McMenamin, Michael Goblirsch

**Affiliations:** 1 United States Department of Agriculture, Agricultural Research Service, Honey Bee Breeding, Genetics, and Physiology Research, Baton Rouge, LA, 78020, USA; 2 United States Department of Agriculture, Agricultural Research Service, Thad Cochran Southern Horticultural Laboratory, Poplarville, MS, 39470, USA

**Keywords:** acute bee paralysis virus, AmE-711, cytopathic effect (CPE), deformed wing virus, host–virus interaction, p.f.u., positive-strand RNA virus (+ssRNA)

## Abstract

Viral pathogens are highly prevalent threats to our pollination workforce. Basic virological techniques for ascertaining infection intensity and quantifying infectious viral particles lag behind the research interest in pollinator-associated viruses. Here, we utilized the only available immortalized honey bee (*Apis mellifera*) cell line, AmE-711, to establish the first plaque assay protocol for a bee-infecting virus in honey bee cells. First, we demonstrate that AmE-711 is susceptible to acute bee paralysis virus (ABPV) despite being persistently infected with deformed wing virus. Then, we compared two traditional overlays, agarose and carboxymethyl cellulose (CMC) and found that the CMC method was superior in producing countable plaques in ABPV-infected monolayers. Lastly, we demonstrated that this method was useful in quantifying infectious particle loads in lysates derived from whole honey bee pupae infected with ABPV, a field-relevant sample type.

## Data availability

Data are available upon reasonable request.

## Code availability

R software code and packages used in statistical analyses are available upon reasonable request.

## Introduction

Pathogenic infections pose a major risk to global food security by both directly hindering crop productivity and indirectly threatening pollinators of food crops [1, 2]. Viral infections are of particular interest as disease-causing agents in our pollination workforce due to their high incidence and relative ease of transmission via shared floral resources [3–6]. Though there has been little empirical investigation of the ecosystem effects of these viral infections, they are important contributors to excess deaths of honey bee colonies [7, 8]. Therefore, pollinator scientists need techniques for incisive and accurate measurement of viruses.

Research into bee-associated viruses has largely been limited to PCR and sequencing-based approaches. While highly sensitive and specific, these nucleic acid detection-based approaches have some notable limitations. First, detection of viral nucleic acids is insufficient evidence of infectious viral particles or a bona fide infection. While efforts have been made to ameliorate this limitation by specifying detection of replicative intermediates, careful experimental follow-up is required to demonstrate productive infection [9]. Recently, this problem has been directly addressed by demonstrating that a novel virus, Andrena-associated bee virus-1, identified in wild bee samples by next-generation sequencing could productively infect primary tissue cultures composed of honey bee pupal cells [5]. Secondly, PCR quantitation of viral nucleic acids has limited utility in estimating true infection intensity, as positive detections can originate from genomes, anti-genomes, transcripts and defective genomes. In fact, there is significant biological variation in the proportion of infectious viral particles to these different nucleic acid species [10].

Applications involving the use of primary cultures and continuous cell lines have proven invaluable for identifying and understanding the role of molecular and cellular processes in organismal health and how disruption of these processes, through infections with pathogens, contributes to disease. For example, the ability to cultivate viruses in cell-based systems has led to the diagnosis and characterization of viral diseases from isolates collected from infected individuals, as well as the development of effective antiviral agents [11, 12]. Furthermore, cell culture methods enable accessible and reliable estimation of infectious viral particles. One such method is the tried-and-true plaque assay in which an infected monolayer of cells is overlaid with a polymer matrix that limits viral particle diffusion, resulting in countable ‘cigarette burns’ or plaques; each plaque effectively denoting an infectious viral particle and referred to as a p.f.u. [13, 14]. While the plaque assay has been applied in bee research using model viruses, standardization of this method for bee-infecting viruses using bee cells has not yet been established [15, 16].

To address a deficiency in our understanding of honey bee–virus interactions, the primary goal of this study was to adapt the viral plaque assay method for use with host-specific AmE-711 honey bee cells. Infectious acute bee paralysis virus (ABPV) produced from either AmE-711 cell cultures or honey bee pupae experimentally infected with ABPV served as the inoculum. By counting the number of PFUs in AmE-711 cell monolayers after infection, we were able to directly quantify infectious ABPV particles in the inoculum. The method was verified by measuring p.f.u. in relation to genome equivalents in infected pupae to simulate field-relevant samples. Therefore, this assay is useful for quantification of infectious virus present in simulated field samples.

## Methods

### Cell culture and virus infection

The AmE-711 honey bee cell line was obtained through an agreement with the University of Minnesota. Since the initial reporting on the establishment of the cell line [17], AmE-711 has been adapted to be grown in commercially available Schneider’s Insect Medium with l-glutamine and sodium bicarbonate (MilliporeSigma) supplemented with 10% heat-inactivated FBS (MilliporeSigma). The cell line is routinely maintained in 75 cm^2^ non-vented flasks (Corning) in a non-humidified incubator at 32 ℃. When cells are to be transferred into a microplate for use in assays, the culture medium is removed from the parent flask, the cells are washed with 1× Dulbecco’s dPBS (dPBS) and the wash is discarded, and 1 ml of 0.25% trypsin-EDTA (ThermoFisher Scientific) is evenly spread over the cells. To facilitate detachment from the flask substrate, the cells are incubated in the presence of trypsin for ~10 min at 32 ℃. Enzymatic activity is halted at the end of the incubation period by adding complete culture medium, the flask is given jarring blows to its sides and the resulting cell suspension is collected and may be passaged through a 40-µm cell strainer (ThermoFisher Scientific) if there is considerable cell clumping. Depending on the number of microplates needed to conduct an assay, cells from several flasks may be pooled.

The ABPV inoculum used for experiments reported here was acquired with permission from the University of Minnesota and originated as a contaminant of a flask of AmE-711 cells. The virus is propagated by exposing a flask containing healthy AmE-711 cells to the inoculum overnight, and the following day, the infectious medium is collected and passaged through a 0.2-µm syringe filter (cellulose acetate, VWR) and stored at −80 ℃ until use. As AmE-711 is persistently infected with deformed wing virus (DWV) [18], the inoculum contains both ABPV and DWV; however, infection with ABPV is responsible for the cytopathic effect (CPE) and DWV load remains relatively constant when the inoculum is added to the cells.

### Honey bee pupal infection and preparation for inoculation

During preliminary experiments, it was observed that FBS-containing medium elicited a strong melanization reaction and subsequent necrosis when injected into white-eyed honey bee pupae. Therefore, to prepare cultures for inoculum production, confluent AmE-711 cells in 75 cm^2^ non-vented flasks (Corning) were washed with 1× dPBS, and FBS-free Schneider’s Insect Medium was added to the flask. Cultures were incubated at 32 ℃ for 24 h to allow the culture to recover and condition the medium. Then, cultures were infected with 10 µl of undiluted, previously prepared ABPV inoculum and returned to the incubator. The next morning, a productive infection was confirmed visually by CPE and near-total cell detachment. The ABPV-rich medium was removed from the flask and freeze-thawed three times to lyse any remaining cells. The infectious medium was then clarified by centrifugation at 4 ℃ at 10,000 RCF for 10 min. The supernatant was then carefully removed so as not to disturb the pellet containing cell debris, then passed through a 0.2-µm syringe filter and stored at −80 ℃ until use. Total RNA was isolated from 100 µL of inoculum by Trizol (ThermoFisher Scientific) following the manufacturer’s instructions, and viral nucleic acids were quantified with ABPV-specific primers by reverse transcription quantitative PCR (see the ‘Virus nucleic acid quantification using reverse transcription quantitative PCR’ section). The inoculum was prepared for injection by serial dilution in dPBS to 10^2^ ABPV genome equivalents (GE) per microlitre.

On day 0 of the experiment, apparently healthy honey bee colonies with no detectable *Varroa destructor* mites by the alcohol wash method [19] were identified as sources of pupae. Frames containing wax-capped pupae were removed from these colonies and brought into the lab. White-eyed pupae were collected from their cells as previously described [20]. First, the wax capping was removed from sealed pupal cells, and then, the pupae were carefully removed by grasping them between the head and thorax with featherweight forceps. Pupae were then placed in a 32 ℃ incubator overnight, and those with visibly melanized wounds were discarded the next day. Healthy, undamaged pupae were injected dorsally between the third and fourth abdominal segments with 2 µl of prepared inoculum for a total dose of 200 ABPV GE or sham-inoculated with dPBS as a control and returned to the incubator. Pupae were snap-frozen on dry ice at 0 and 48 h post-infection (hpi) and stored at −80 ℃ until use.

To prepare pupae for inoculation of subsequent pupae or confluent AmE-711 cultures, a single pupa was homogenized in 1 ml of sterile PBS. The lysate was then clarified by centrifugation at 10,000 RCF for 10 min at 4 °C and passed through a 0.22-µM PVDF syringe filter (Millipore) to remove debris and microbes. The resulting inoculum was then stored at −80 ℃ until use.

### Virus nucleic acid quantification using reverse transcription quantitative PCR

Quantity and quality of total RNA were assessed by spectrophotometry using a NanoDrop One (ThermoFisher Scientific). One microgram of total RNA was converted into cDNA using LunaScript RT SuperMix (New England Biolabs) following the manufacturer’s instructions. One microlitre of the RT reaction was used directly in a 20 µl quantitative PCR (qPCR) reaction using Luna Universal qPCR Master Mix and 0.25 µM each of ABPV-specific primers [21] (F: 5′-ACC GAC AAA GGG TAT GAT GC-3′, R: 5′-CTT GAG TTT GCG GTG TTC CT-3′) or DWV-specific primers [22] (F: 5′-GAG ATT GAA GCG CAT GAA CA-3′, R: 5′-TGA ATT CAG TGT CGC CCA TA-3′). A linear plasmid standard containing the target amplicons (synthesized by IDT) was included during the reaction, and GE was estimated by interpolation of the calculated standard curve (ABPV: *R*^2^=0.9965, efficiency=86.64%; DWV: *R*^2^=0.9979, efficiency=91.29%).

### Endpoint dilution assay

Prior to conducting the plaque assay with infected pupal tissue, the range of working dilutions was empirically determined by endpoint dilution assay [23]. To do this, AmE-711 honey bee cells were seeded at an approximate density of 1.0×10^5^ cells per well of a 96-well flat-bottom Nunclon Delta Surface tissue culture-treated microplate (ThermoFisher Scientific) using 100 µl of complete medium per well. The cells were allowed to recover from exposure to trypsin and adapt to the microplate environment for 2 full days before use in the assay. On the third day after seeding, a tenfold serial dilution of the inoculum was prepared in FBS-free Schneider’s Insect Medium, spanning the range from 10^−1^ to 10^−11^. To infect the cells, the culture medium was removed from the wells using a multichannel pipette, the cells were washed with 100 µl of 1× dPBS and the wash was discarded, and then 100 µl of each virus dilution was added per well to a column of eight replicate wells of the microplate. A column of eight wells was designated as the mock-infected control, which consisted of conditioned culture medium from a flask of healthy AmE-711 cells persistently infected with DWV. Cells were incubated for 1 h at 32 ℃ in the presence of the inoculum to allow adsorption of viral particles. At the end of the incubation period, the inoculum was removed, and the cells were again washed with 100 µl of 1× dPBS and the wash was discarded, which was then followed by the addition of 100 µl complete medium to each well. After which, the microplate was returned to the incubator for 24 h. The following day, each well was scored for the presence of CPE (1=CPE observed and 0=CPE not observed). A plaque assay was then run in 6-well plate format with the following: [1] the inoculum dilution that produced CPE in 4±1 wells of 8 [2], two serial dilutions above the ~50% CPE dilution [3], two serial dilutions below the ~50% CPE dilution and [4] a negative control. In practice, these might be the following six wells: 10^−6^, 10^−7^, 10^−8^, 10^−9^, 10^−10^ and a negative control.

### Viral plaque assay

AmE-711 cells were seeded at an approximate density of 2.0–2.5×10^6^ cells per well of a 6-well flat-bottom tissue culture-treated microplate (Corning) using 1–2 ml of complete medium per well. Prior to the addition of cells, each well was pre-equilibrated with 1 ml of complete culture medium. Cells were allowed to recover from trypsin exposure and adapt to the microplate environment for 2 full days prior to running the assay and were visually inspected using light microscopy to ensure they had formed a dense, confluent monolayer. At the start of the assay, a tenfold serial dilution of the inoculum was prepared, spanning the range from 10^−6^ to 10^−10^. One well of the plate was devoted to the negative control, which consisted of conditioned culture medium from a flask of healthy AmE-711 cells persistently infected with DWV.

At the start of the assay, the culture medium was removed from each well and the cells were washed with 1× dPBS and discarded. Next, 1 ml of each dilution of inoculum was added to the wells. The inoculated plate was returned to the incubator for 1 h at 32 ℃ to allow adsorption of viral particles. Every 15 min during the incubation period, the plate was removed from the incubator briefly, and the inoculum was redistributed across the cells to ensure homogeneous dispersion of viral particles. At the end of the incubation period, the inoculum was removed from the wells, and the cells were again washed with 1 ml of 1× dPBS. Immediately following, 4 ml of overlay was gently added to each well. Preliminary experiments were conducted to optimize the type and concentration of overlay that would reproducibly permit its efficient removal from the wells without damaging the underlying cell layers and yield countable plaques of uniform size.

A standard solid agarose overlay and a semisolid carboxymethyl cellulose (CMC) overlay were tested, similar to Mendoza *et al*. [24]. Stock solutions (3% w/v) of both overlays were prepared by dissolving either ultrapure low-melting-point agarose (ThermoFisher Scientific) or CMC medium viscosity sodium salt (MilliporeSigma) in cell- and molecular-grade sterile water at 121 ℃ for 30 min. The overlay stock solutions were maintained at 47 ℃ until use. A 1 : 3 dilution of 3% CMC was prepared within 15 min prior to the end of the viral inoculation period, and a 1 : 9 dilution of 3% agarose was prepared immediately before the removal of the inoculum from the wells, both in FBS-free Schneider’s Insect Medium. The overlay was allowed to cool for up to 30 min at room temperature before the plate was carefully transferred to a 32 ℃ incubator.

After 24 h, the overlay was removed from the wells by tilting the plate and allowing it to collect into a receptacle designated for disposal as biohazardous waste. Any residual overlay was removed with a micropipette. One millilitre of 4% buffered formaldehyde solution, pH 6.9 (MilliporeSigma), was added to each well, and the cells were fixed for 1 h at room temperature. The fixative was then removed, and 1 ml of 1% aqueous crystal violet solution (MilliporeSigma) was added to each well, and the cells were stained for 1 h at room temperature. When the staining period was finished, the crystal violet solution was removed, and the wells were washed one or two times with 1 ml of sterile water per wash to make visualization of plaques for counting easier. The dilution of inoculum that yielded 5–100 plaques per well was used in a second assay that consisted of six replicate wells exposed to this dilution. Plaques were enumerated from the six wells, and the mean number of plaques was converted to p.f.u.ml^−1^ according to the following formula:

Formula 1.


$$PFU/mL=\frac{MeanNumberofPlaquesfrom6ReplicateWells}{DilutionFactor*VolumeofInoculum(mL)}$$

To compare the variation in plaque size between the two overlays, a 22×22 mm coverslip was placed in each of the six wells per overlay. Plaques were then observed using a SZX9 stereo microscope connected to a UC90 digital camera (Olympus). The diameter of each plaque under the coverslip was measured using the arbitrary line tool found in the cellSens Standard imaging software (Olympus).

### Statistical analysis

Statistical analyses and data visualization were performed in R version 4.4.0 (‘Puppy Cup’) [25]. Group differences in PFU or GE were analysed by the lm() function in the base R package *stats*. Data were visualized using the *ggplot2* package with minor aesthetic edits in Adobe Illustrator v 26.2.1.

## Results

### AmE-711 is permissive to ABPV infection

**Fig. 1.**
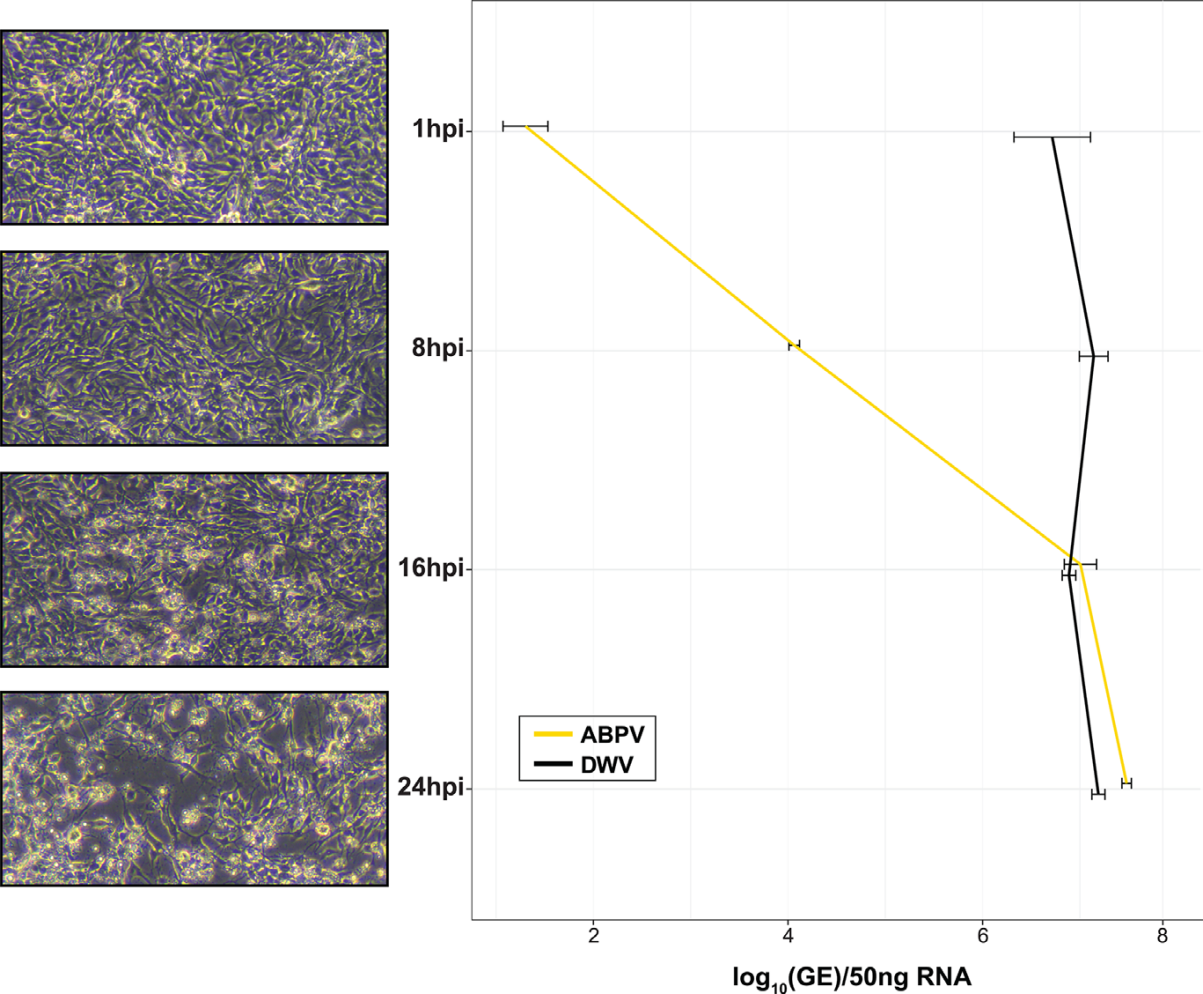
AmE-711 cells infected with ABPV. Representative microscopic images (100×) of AmE-711 cells infected with ABPV were captured at 1, 8, 16 and 24 hpi (left). The increase in CPE with time of ABPV infection corresponded to the increase in ABPV genome equivalents (GE) (yellow line) detected in total RNA extracted from AmE-711 cells at the specified timepoints (right). Persistent infection of AmE-711 with DWV (black line) remained stable throughout the time course of ABPV infection.

First, the permissibility of AmE-711 to infection by ABPV was established by examination of CPE and quantification of viral RNA following exposure (Fig. 1). Representative images taken of AmE-711 cells at 1, 8, 16 and 24 h after inoculation with ABPV show increased numbers of cells undergoing lysis over time, indicating a productive infection. Furthermore, reverse transcription qPCR on samples collected at the four timepoints of infection demonstrates ABPV nucleic acids increased with severity of CPE. At 1 hpi, ABPV GE fell below our limit of detection but increased to an average of 3.022×10^7^±3.59×10^6^ GE by 24 hpi. In contrast, DWV GE were static throughout the infection period, varying between 5.19×10^6^ and 1.55×10^7^. Moreover, the DWV GE for any timepoint was no different than that at one hpi (simple linear regression, *F*_3,8_=1.067, *P*=0.416).

### Comparison of plaque formation using agarose vs. carboxymethyl cellulose overlays

**Fig. 2.**
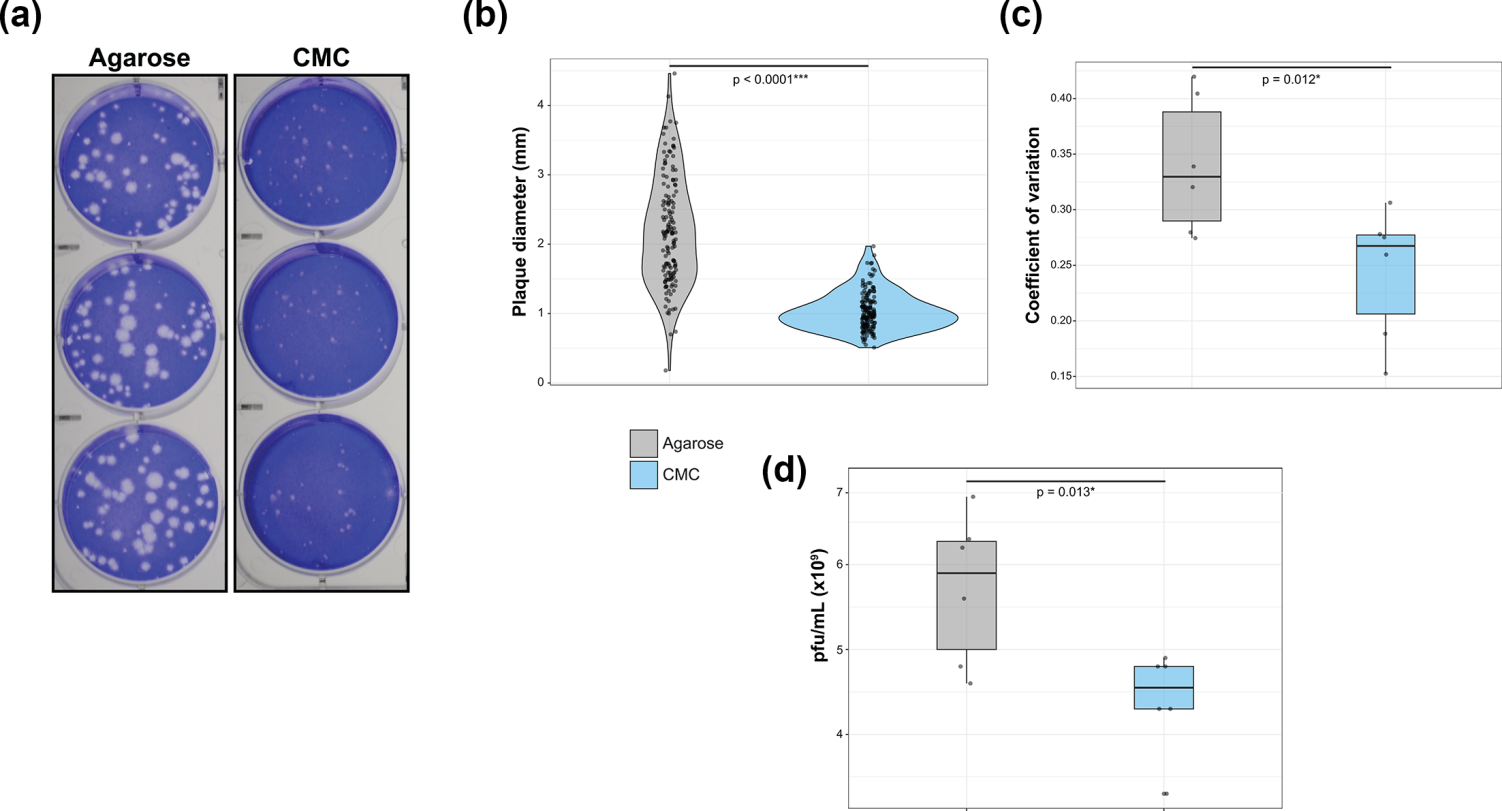
Comparison of plaque formation using agarose vs. CMC overlays on AmE-711 cells infected with ABPV. (**a**) Three replicate wells of each overlay, demonstrating differences in plaque size and morphology between the two overlays. Acute bee paralysis virus-infected AmE-711 cells were fixed with 4% formaldehyde and stained with 1% crystal violet solution prior to imaging. (**b**) There was a significant difference in the distribution of plaque diameter (mm) (multiple linear regression, estimate=−1.16 mm, *F*_2,283_=142.9, adjusted *R*^2^=0.499, *P*<2.2×10^−16^) and (**c**) variability in size among plaques between the two overlays (estimate=−9.6%, *F*_2,9_=7.6, adjusted *R*^2^=0.545, *P*=0.0117). (**d**) The CMC overlay resulted in significantly fewer countable plaques relative to the agarose overlay (SLR, estimate=1.32×10^9^ p.f.u. ml^−1^, *F*_1,10_=9.21, adjusted *R*^2^=0.43, *P*=0.013).

There were notable differences in plaque formation between the two overlays. Plaque morphology was far more variable visually when the agarose overlay was employed as compared to the CMC overlay (Fig. 2a). On average, the CMC overlay resulted in smaller, more punctate plaques (Fig. 2b, multiple linear regression, estimate=−1.16 mm, *F*_2,283_=142.9, adjusted *R*^2^=0.499, *P*<2.2×10^−16^) that were more uniform in plaque size and had a smaller coefficient of variation (Fig. 2c, estimate=−9.6%, *F*_2,9_=7.6, adjusted *R*^2^=0.55, *P*=0.012), but also more numerous (Fig. 1d) when the agarose overlay was used (SLR, estimate=1.32×10^9^ p.f.u. ml^−1^, *F*_1,10_=9.21, adjusted *R*^2^=0.43, *P*=0.013).

### Genome equivalents may outnumber infectious viral particles nearly tenfold

**Fig. 3.**
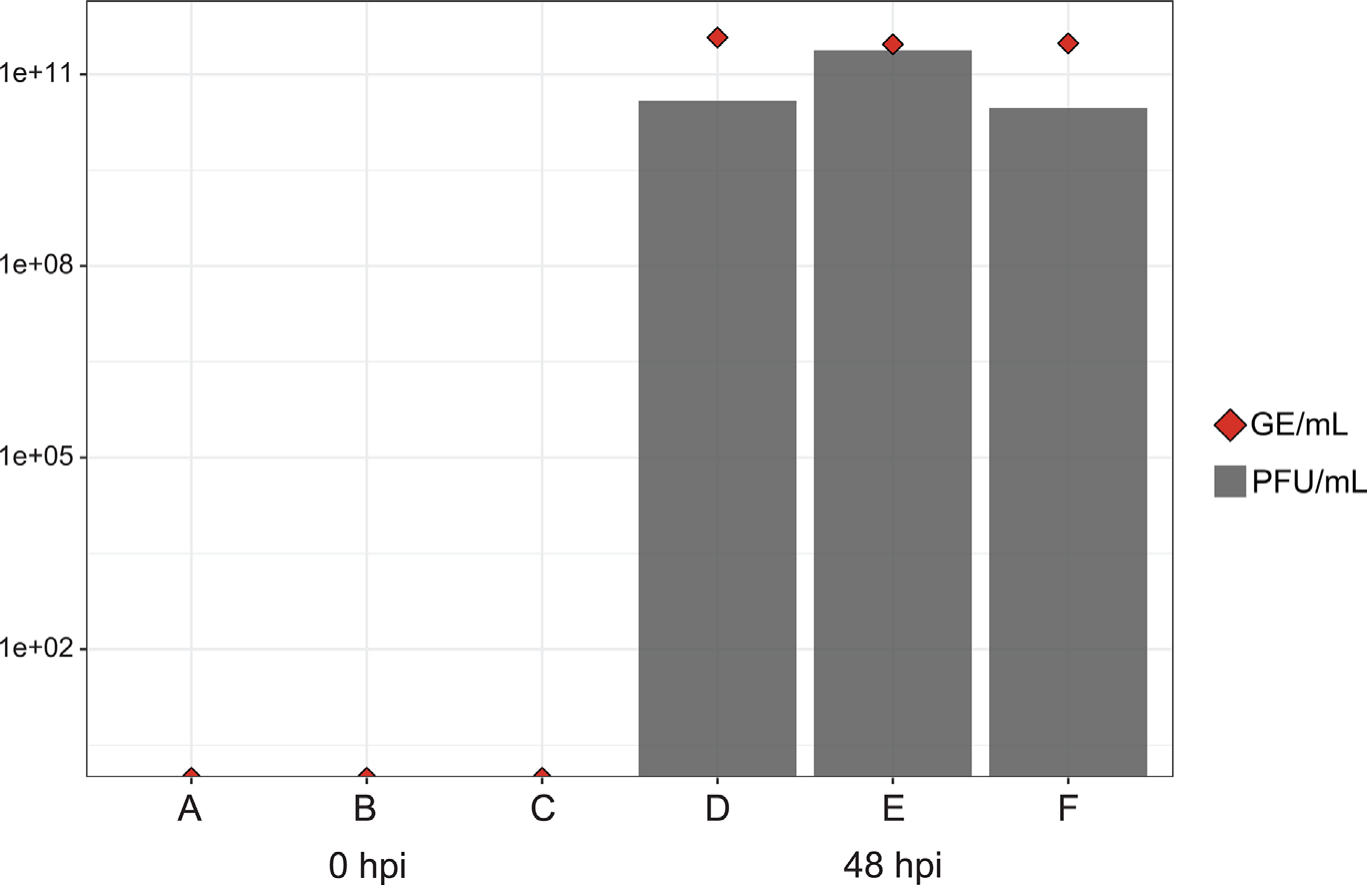
Comparison of GE to p.f.u. per millilitre of sample containing ABPV that was produced in experimentally infected, white-eyed honey bee pupae. Genome equivalents (red diamonds) were quantified by quantitative reverse transcription PCR from total RNA extracted from infected pupae using ABPV-specific primers, and p.f.u. (grey bars) were estimated by plaque assay using carboxymethyl cellulose overlay on ABPV-infected AmE-711 cells. Three replicate pupae were sampled at 0 (**a–c**) and 48 (**d–f**) hpi.

To test the suitability of the plaque assay on field-relevant samples, honey bee pupae were injected with 100 ABPV GE or sham-injected with PBS and then collected at 0 and 48 hpi. By 72 hpi, all ABPV-injected pupae were necrotized and assumed dead. Then, reverse transcription qPCR and CMC-overlay plaque assays were used in parallel to measure GE and infectious viral particles, respectively. None of the PBS-injected pupal lysates had detectable ABPV nucleic acids or resulted in plaque formation. At 0 hpi, virus-injected pupae similarly had no detectable ABPV by either assay (Fig. 3). By 48 hpi, pupae had 2.96×10^11^ to 3.79×10^11^ GE and 3.87×10^10^ to 2.40×10^11^ p.f.u. per 1 ml of lysate, a roughly 10 : 1 ratio of GE/p.f.u. The notable exception was replicate B at 48 hpi, which had lower GE and higher PFU than either sample A or C.

## Discussion

Empirically determining optimal conditions for a given cell type-virus pair is a crucial step in establishing a utilizable plaque assay. Indeed, culturing conditions and overlay type show considerable variation in resultant plaque count and morphology depending on the virus in question [26]. Moreover, one must first establish a cell type that is permissive to infection by the virus of interest and that potentially confounding persistent infections do not also result in cytopathic effects or affect plaque formation. There is also the consideration of plaque morphology, as a large variation in plaque size or diffuse plaque borders may result either due to suboptimal conditions or perhaps due to biophysical interactions of the matrix itself with the virions. Here, we established that AmE-711 cells are highly permissive to ABPV infection. And, while both agarose and CMC overlays have apparent utility, CMC is likely the better choice for producing more consistent and countable resultant plaques in the AmE-711 cell culture system. Importantly, CPE and plaque formation occurred in the presence of persistent DWV infection, but not in the absence of ABPV. Thus, persistent DWV infection does not apparently affect ABPV plaque formation in this culturing system.

Furthermore, we demonstrate that the plaque assay with a CMC overlay is compatible with sterile-filtered lysates generated from field-relevant samples. Live pupae were injected with ABPV, which replicated from undetectable levels at 0 hpi to more than 10^10^ GE and p.f.u. at 48 hpi (Fig. 3), indicating a productive infection. As expected, GE outnumbered PFU, as nucleic acids necessarily outnumber infectious viral particles. We observed a roughly 10 : 1 ratio of GE to p.f.u. in these samples. This ratio and its between-sample variation are comparable to HeLa S3 cells infected with Poliovirus Mahoney type I at a low multiplicity of infection [10]. Therefore, we conclude that our assay conditions were suitable for whole organism-derived samples.

The applicability of a plaque assay versus quantitative reverse transcription (qRT)-PCR depends a great deal on the question of interest and the experimental setup being used. Undoubtedly, qRT-PCR is a more rapid, accessible and sensitive method for quantifying viral nucleic acids and interpolating viral load from a sample. Establishing viral load through qRT-PCR suffices for most studies; however, there are cases where one might need a measure of actual infectivity. For example, reproducible *in vitro* pharmaceutical screening for molecules with putative antiviral activity requires precise measurement of infectious virus used for inocula. This is because one wants to be sure that the multiplicity of infection at the start of the experiment is consistent between inoculum sources across experiments. Furthermore, given significant biological noise in the relative proportions of viral particles and viral nucleic acid species, even within highly homogenous immortalized cell cultures [10], it is not unlikely that host species has a deterministic impact on these relative proportions as well. Therefore, in the expanding field of comparative virology between pollinator species for the purposes of evolutionary studies and biopesticide risk assessments, accurate measures of infectious viral particles will become more important.

## Conclusions

Here, we present a method for direct quantification of infectious viral particles of ABPV, a commonly detected virus with implications for hive longevity [27]. Importantly, this method utilizes an immortalized cell line established from honey bee tissues, increasing the utility and biological relevance in studies of bee-infecting viruses. For viruses that are not cytopathic, this cell line could conceivably be used for quantification of infectious viral particles using more modern methods like fluorescent immunofocus assays [28].

## Abbreviations

ABPV: acute bee paralysis virus
CMC: carboxymethyl cellulose
CPE: cytopathic effect
dPBS: Dulbecco’s PBS
DWV: deformed wing virus
GE: genome equivalents
hpi: hours post infection
qPCR: quantitative PCR
qRT: quantitative reverse transcription

## References

[1] Sundström JF, Albihn A, Boqvist S, Ljungvall K, Marstorp H (2014). Future threats to agricultural food production posed by environmental degradation, climate change, and animal and plant diseases – a risk analysis in three economic and climate settings. *Food Sec*.

[2] Page ML, Davis JK, Glasser SK, Kusi EO, Lindsay SE (2025). Mechanisms and consequences of plant–pollinator–pathogen interactions. Annu Rev Ecol Evol Syst.

[3] Alger SA, Burnham PA, Brody AK (2019). Flowers as viral hot spots: Honey bees (Apis mellifera) unevenly deposit viruses across plant species. PLoS One.

[4] Deutsch KR, Graham JR, Boncristiani HF, Bustamante T, Mortensen AN (2023). Widespread distribution of honey bee-associated pathogens in native bees and wasps: trends in pathogen prevalence and co-occurrence. J Invertebr Pathol.

[5] Daughenbaugh KF, Kahnonitch I, Carey CC, McMenamin AJ, Wiegand T (2021). Metatranscriptome analysis of sympatric bee species identifies bee virus variants and a new virus, andrena-associated bee virus-1. Viruses.

[6] Kahnonitch I, Daughenbaugh KF, Arkin N, Erez T, Dorchin A (2025). Virus distributions in wild bees are associated with floral communities at local to landscape scales. Ecol Appl.

[7] Nearman A, Crawford CL, Guarna MM, Chakrabarti P, Lee K (2025). Insights from U.S. beekeeper triage surveys following unusually high honey bee colony losses 2024-2025. Sci Total Environ.

[8] McMenamin AJ, Genersch E (2015). Honey bee colony losses and associated viruses. Curr Opin Insect Sci.

[9] Posada-Florez F, Childers AK, Heerman MC, Egekwu NI, Cook SC (2019). Deformed wing virus type A, a major honey bee pathogen, is vectored by the mite Varroa destructor in a non-propagative manner. Sci Rep.

[10] Schulte MB, Andino R (2014). Single-cell analysis uncovers extensive biological noise in poliovirus replication. J Virol.

[11] Guo Y, Goodman CL, Stanley DW, Bonning BC (2020). Cell lines for honey bee virus research. Viruses.

[12] Atampugbire G, Adomako EEA, Quaye O (2025). *In vitro* antiviral assays: a review of laboratory methods. ASSAY Drug Dev Technol.

[13] Cooper PD, Smith KM, Lauffer MA (1962). Advances in Virus Research.

[14] Dulbecco R (1952). Production of plaques in monolayer tissue cultures by single particles of an animal virus. Proc Natl Acad Sci U S A.

[15] Flenniken ML, Andino R (2013). Non-specific dsRNA-mediated antiviral response in the honey bee. PLoS One.

[16] Brutscher LM, Daughenbaugh KF, Flenniken ML (2017). Virus and dsRNA-triggered transcriptional responses reveal key components of honey bee antiviral defense. Sci Rep.

[17] Goblirsch MJ, Spivak MS, Kurtti TJ (2013). A cell line resource derived from honey bee (*Apis mellifera*) embryonic tissues. PLoS One.

[18] Carrillo-Tripp J, Dolezal AG, Goblirsch MJ, Miller WA, Toth AL (2016). In vivo and in vitro infection dynamics of honey bee viruses. Sci Rep.

[19] De Jong D, De Andrea Roma D, Gonçalves LS (1982). A Comparative analysis of shaking solutions for the detection of varroa jacobsoni on adult honeybees. Apidologie.

[20] McMenamin AJ, Parekh F, Lawrence V, Flenniken ML (2021). Investigating virus-host interactions in cultured primary honey bee cells. Insects.

[21] Vanengelsdorp D, Evans JD, Saegerman C, Mullin C, Haubruge E (2009). Colony collapse disorder: a descriptive study. PLoS One.

[22] Lanzi G, de Miranda JR, Boniotti MB, Cameron CE, Lavazza A (2006). Molecular and biological characterization of deformed wing virus of honeybees (*Apis mellifera* L.). J Virol.

[23] Reed LJ, Muench H (1938). A simple method of estimating fifty per cent endpoints12. Am J Epidemiol.

[24] Mendoza EJ, Manguiat K, Wood H, Drebot M (2020). Two detailed plaque assay protocols for the quantification of infectious SARS-CoV-2. Curr Protoc Microbiol.

[25] Team RC (2024). R: A Language and Environment for Statistical Computing. 4.1.2 ed.

[26] Baer A, Kehn-Hall K (2014). Viral concentration determination through plaque assays: using traditional and novel overlay systems. J Vis Exp.

[27] Lamas ZS, Rinkevich F, Garavito A, Shaulis A, Boncristiani D (2025). Viruses and vectors tied to honey bee colony losses. bioRxiv.

[28] Counihan NA, Daniel LM, Chojnacki J, Anderson DA (2006). Infrared fluorescent immunofocus assay (IR-FIFA) for the quantitation of non-cytopathic and minimally cytopathic viruses. J Virol Methods.

